# Virtual Reality Simulator versus Conventional Advanced Life Support Training for Cardiopulmonary Resuscitation Post-Cardiac Surgery: A Randomized Controlled Trial

**DOI:** 10.3390/jcdd10020067

**Published:** 2023-02-04

**Authors:** Jette J. Peek, Samuel A. Max, Wouter Bakhuis, Isabelle C. Huig, Rodney A. Rosalia, Amir H. Sadeghi, Edris A. F. Mahtab

**Affiliations:** 1 Department of Cardiothoracic Surgery, Erasmus MC, University Medical Center Rotterdam, 3015 GD Rotterdam, The Netherlands; 2 Medical Sciences Division, University of Oxford, Oxford OX1 2JD, UK; 3 Department of Anesthesiology, Erasmus MC, University Medical Center Rotterdam, 3015 GD Rotterdam, The Netherlands

**Keywords:** cardiac surgery, cardiopulmonary resuscitation, emergency resternotomy, virtual reality, simulation training, manikin training, medical training, virtual reality simulation

## Abstract

External chest compressions are often ineffective for patients arresting after cardiac surgery, for whom emergency resternotomy may be required. A single-blinded randomized controlled trial (RCT) was performed, with participants being randomized to a virtual reality (VR) Cardiac Surgical Unit Advanced Life Support (CSU-ALS) simulator training arm or a conventional classroom CSU-ALS training arm. Twenty-eight cardiothoracic surgery (CTS) residents were included and subsequently assessed in a moulage scenario in groups of two, either participating as a leader or surgeon. The primary binary outcomes were two time targets: (1) delivering three stacked shocks within 1 min and (2) resternotomy within 5 min. Secondary outcomes were the number of protocol mistakes made and a questionnaire after the VR simulator. The conventional training group administered stacked shocks within 1 min in 43% (n = 6) of cases, and none in the VR group reached this target, missing it by an average of 25 s. The resternotomy time target was reached in 100% of the cases (n = 14) in the conventional training group and in 83% of the cases (n = 10) in the VR group. The VR group made 11 mistakes in total versus 15 for those who underwent conventional training. Participants reported that the VR simulator was useful and easy to use. The results show that the VR simulator can provide adequate CSU-ALS training. Moreover, VR training results in fewer mistakes suggesting that repetitive practice in an immersive environment improves skills.

## 1. Introduction

The cardiopulmonary resuscitation (CPR) protocol for post-cardiac surgery patients differs from the generic advanced life support protocol for other clinical patients [1,2,3]. Principal differences of the Cardiac Surgical Unit Advanced Life Support (CSU-ALS) guidelines include the immediate delivery of three consecutive external defibrillation shocks in case of a shockable rhythm, early pacing when asystole or severe bradycardia is witnessed, and decompression of tension pneumothorax, where up to a one-min delay in starting basic life support is permitted. Other discrepancies include early emergency resternotomy, which should be performed in less than 5 min if indicated, and caution and/or reduced doses when administering epinephrine (typically 0.1–0.25 mg instead of 1 mg) [1,2]. Several studies have shown that training and practicing based on a structured protocol improve the time to recognize the need for resternotomy and the time to reopen the thorax [2,4]. External chest compressions are ineffective for most causes of cardiac arrest after cardiac surgery (e.g., hypovolemia, cardiac tamponade, tension pneumothorax). Consequently, emergency resternotomy within five mins reduces complications and can improve patient outcomes [2,4].

Adherence and prompt progression through these guidelines have been shown to improve outcomes in patients who experience cardiac arrest after cardiac surgery and expedite the decision and execution of a resternotomy [2]. However, the relative infrequency of these events limits the exposure and impedes knowledge retention of the CSU-ALS protocol for CTS trainees and allied healthcare staff who care for post-cardiac surgery patients. Currently, the gold standard for CPR education is a certified Advanced Life Support (ALS) training, based on guidelines by the European Resuscitation Council (ERC) [5]. A blended approach is often employed for these ALS training courses, including theoretical learning and skills training, combining reading a manual, e-learning, presentations, or webinars with hands-on training such as scenario simulation with manikins and reflection with the tutor and group [5]. Knowledge and skills are then evaluated by certified instructors in a moulage environment. Participants are assessed on their adherence to the ALS protocol, after which they receive certification. However, interactive and immersive learning environments that are independent of classroom facilities and ALS instructors still need to be made available.

A virtual reality (VR) simulation (cardiopulmonary virtual reality simulator, CPVR-sim) was developed previously by our group to train physicians and members of the multidisciplinary team for several post-cardiac surgery CPR scenarios [6]. In this simulation, the user can practice scenarios repeatedly and learn the steps described within the protocol in a realistic, 360 degree, immersive, 3D environment without the need for other equipment (aside from a VR headset and controllers) or instructor-led training sessions. At present, this simulator’s face and content validity have been demonstrated in a feasibility study, in which experts and their junior colleagues alike found this training method useful, easy to use, and suitable as an adjunct to conventional CPR training [6]. Such gamified learning can be used in ALS training for applications such as self-study before an ALS course, in a blended learning approach, and for learning without being dependent on the time and location of an instructor and materials [5]. The ERC guidelines suggest that ALS skills should be taught in several distinct ways, including apportioning steps relating to a particular skill into a realistic simulation, explanation of theory, demonstration, and practicing by trainees [5]. The aforementioned teaching methods are included in the CPVR-sim by virtue of the step-by-step guidance, explanation of theory and protocol guidelines, and the unlimited hands-on practice in a realistic VR environment.

In order to assess the concurrent validity and effectiveness of the CPVR-sim, we performed the first randomized controlled trial (RCT) to show the utility of the CPVR-sim compared with the gold standard of CSU-ALS training. The goal was to impart the key differences between the standard ALS guidelines versus the CSU-ALS guidelines, the components of international guidelines, and other clinical insights regarding the management of post-cardiac surgery arrests [3].

We aimed to show that participants who undergo VR training can progress through the CSU-ALS algorithm accurately and as quickly as participants who undertake the traditional training, thereby demonstrating the utility of the CPVR-sim as a replacement for current training protocols. This randomized trial will therefore compare the timing of predefined clinical endpoints and technical steps, as well as the accuracy with which the algorithm is followed between the control group (gold standard CSU-ALS training) and the VR group (CPVR-sim).

## 2. Methods

The research protocol was approved by the Erasmus Medical Center Medical Ethical Review Committee (MEC-2022-0227). The trial was registered with the International Standard Randomized Controlled Trial Number registry, with ID ISRCTN57122010. The Consolidated Standards of Reporting Trials (CONSORT) guideline was used for reporting this research, and the CONSORT flowchart and checklist can both be found in the Supplementary Material S1 and S2, respectively [7]. This study was carried out on the 19th of May 2022 on a Dutch national teaching day for all cardiothoracic surgery (CTS) residents in training in the Netherlands.

Participants were all CTS residents in the Netherlands who had at least 1 year of experience in cardiothoracic surgery and who provided informed consent. Participants were excluded if they were not able to partake in the conventional or VR training, or were not able to perform the physical assessment. They first filled in a baseline questionnaire (Supplementary Materials S3) about their work experience in the field of CTS, cardiac arrest in patients after cardiac surgery, and (emergency) resternotomies.

### 2.1. Randomization and Blinding

Castor Electronic Data Capture version 2022.2.2 (Ciwit B.V., Amsterdam, the Netherlands) was used for data collection and randomization. For this 1:1 non-crossover RCT, we used stratified block randomization with a block size of 4, distributed according to work experience using a binary cutoff at four years of work experience. This was performed to ensure the work experience in both groups was distributed equally. Participants and study personnel physically facilitating both trainings were not blinded during this study, as the apparent differences in training delivery methods of the two groups did not allow for this. Study personnel who facilitated the physical assessment and performed data collection and analysis, including the experts who assessed the video recordings, were blinded.

### 2.2. Interventions

All participants were obliged to study the materials provided before starting the course. These materials included: the guidelines for resuscitation of patients who arrest after cardiac surgery [2], a video including different scenarios for cardiac arrest after cardiac surgery, and a video of a resternotomy procedure. Based on reported work experience, the participants were randomized into two groups: the VR group or the control group.

**Figure 1:**
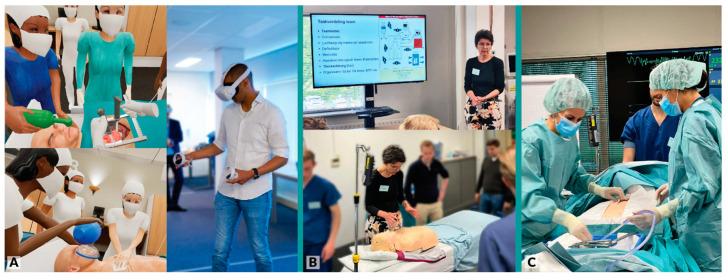
(**A**) Screenshots and pictures of the VR training; (**B**) pictures of the conventional training; (**C**) a participant performing the assessment case using the manikin.

Participants in the VR group first received a short (5 min) introductory briefing on how the VR headset and controllers worked. Next, they performed the CPVR-sim simulation training, including three different cardiac arrest cases, encompassing each of the arms of the CSU-ALS guidelines. The CPVR-sim took approximately 30–45 min per participant, depending on their skills and experience with VR/gaming and their knowledge of the CSU-ALS protocol. Still images of the immersive VR training and selected pictures of the study activities are shown in Figure 1. Participants in the control group received conventional CSU-ALS training from a certified CSU-ALS instructor (Figure 1B). This training included a presentation of the protocol (15 min) and simulation training (45 min) with a resternotomy-manikin (CSU-ALS 4th generation manikin, CALS, United Kingdom).

### 2.3. Study Design

Both initial trainings (control group and VR group) were completed with a standardized physical assessment (role play/moulage), using a resternotomy manikin. The assessment case was the same for all participants to facilitate a direct comparison of participants’ performance (Supplementary Materials S4). Before the assessment started, the participants were divided into pairs within the same randomization group. These pairs were randomly assigned to perform as either the team leader or the surgeon using closed envelopes [2]. Four medical students participated as nurses during the physical assessments to fulfill all key roles in cardiac arrest [2]. These nurses only performed tasks if they were instructed to do so by the team leader or surgeon, and the personnel were the same for all assessments.

The physical assessments were recorded on video and a blinded independent expert assessor evaluated and timed the videotapes, based on a list of key actions, according to the CSU-ALS guidelines (Supplementary Materials S4) [2]. An image of the physical assessment is shown in Figure 1C. For the primary outcome, all participants were timed, and the time was recorded at two points: (1) when the team administered the third of three shocks as instructed by the team leader, and (2) when resternotomy was performed by the surgeon and their assistant.

The guidelines state that basic life support can be postponed for one min whilst stacked shocks or pacing are performed where applicable and that resternotomy should ideally be performed within five mins [2]. Therefore, we employed a time target of one min to deliver the three stacked shocks and a time target for the resternotomy of five mins. The moment of resternotomy was defined as when the sternal retractor was opened, since from that time-point onwards, the surgeons have a full view of the heart, and a tamponade would be relieved. As secondary endpoints, we considered protocol deviations, defined as the incorrect order of actions, missed steps, or incorrect execution of an action. We also compared the time to specific actions that should be performed as stated in the international guidelines.

**Figure 2:**
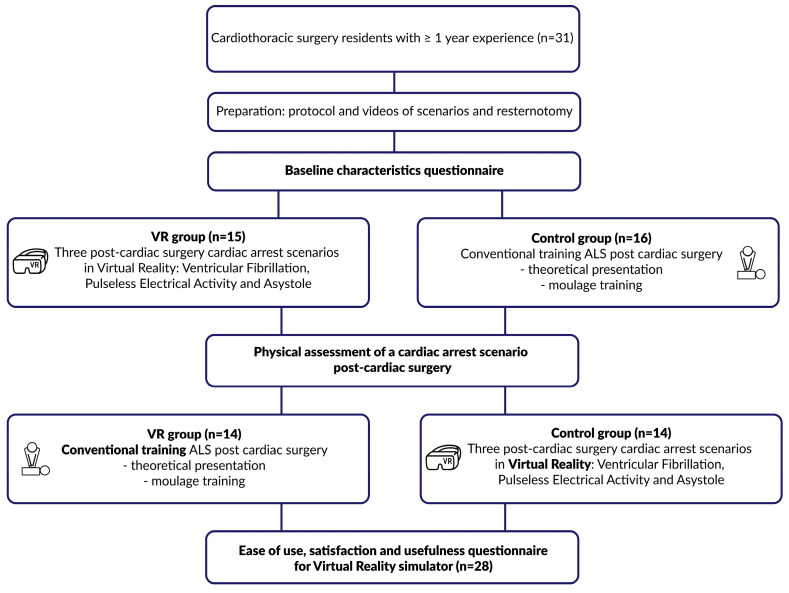
Flowchart of the study design of this randomized control trial.

After the assessments were completed, the groups were switched, and the VR group received conventional training and vice versa, such that eventually, all residents underwent both types of training. Finally, as a secondary endpoint, we assessed the usefulness, satisfaction, and ease of use (USE) of VR training by using objective questionnaires based on the standardized USE questionnaire to be filled out by all participants [8]. A flowchart of the study design can be found in Figure 2.

### 2.4. Statistical Analysis

We proposed that the two methods of training would clinically differ significantly if the time taken to deliver the third shock was more than 12 s above the shock time target (20%) and the time to resternotomy varied by more than 30 s (10%). Therefore, with a power of 0.8, alpha of 0.05, and a beta of 0.2, we calculated that this study required a sample size of 30 participants, approximately equivalent to the total number of CTS residents in the Netherlands.

IBM SPSS version 28 (IBM corp., Armonk, NY, USA) was used for analysis. Continuous data were assessed for normal distribution using a Q–Q plot and Shapiro–Wilk test. Data were reported as median (IQR) or mean ± SD and compared using either an unpaired *t*-test or the Mann–Whitney U test (whichever was appropriate according to the distribution), which were used to determine the significance for the demographic data. Categorical variables were reported as numbers (%) and compared using a χ2 test or Fisher’s exact test (whichever was appropriate according to the distribution). For the time target data, a one-sample *t*-test was performed comparing the means of each group to the two time targets. A *p*-value of <0.05 was considered statistically significant.

### 2.5. Exclusion of Outliers

The data were checked for equal variance amongst both groups for each time target. It was found that the VR group had a variance that was more than twice as large as the control group. Further inspection, and analytical methodologies including Tukey’s Fences test (1.5 interquartile range rule), Grubbs’ test, Dixon’s Q test, and visual inspection using box, scatter, and Q–Q plots revealed a single data point for the resternotomy time as an outlier. Video footage from this case was reviewed, and it was found that the participants had not been given a complete briefing regarding the use of equipment available to them, and hence were delayed in performing a resternotomy due to factors outside the control of the participants. These plots and test values can be found in Supplementary material S5. The outlying data point was excluded from analysis as described above.

## 3. Results

### 3.1. Baseline Characteristics

Of the 40 Dutch residents, 31 registered for the training day, provided informed consent, and were randomized into two groups. In total, three participants were excluded from the study. Two were excluded because one of them participated in the wrong training, resulting in a mixed VR and control group, and an additional one due to an odd number of participants, resulting in one assessment where the researchers assisted in the moulage scenario. Finally, 28 participants were included, and 14 residents were randomized to the VR group and 14 to the control group. The mean age was 31.3 ± 1.9 years and 32.3 ± 2.4 years for the VR and control groups, respectively. The baseline characteristics for the two groups were comparable, as shown in Table 1. After dividing the groups into pairs (team leader and surgeon), there were seven pairs per group performing the assessment.

### 3.2. Primary Outcomes

The primary outcomes were binary and based on (1) performing the third shock within 1 min and (2) reopening the chest within 5 mins.

Six (43%) participants were able to administer stacked shocks within 1 min after conventional training, whereas none of the participants in the VR group (0%) reached this time target (Table 2). The control group was not significantly faster than the 1-min time target, but the VR group was considerably slower. The mean time to deliver the stacked shocks was 62.7 ± 8.9 s and 85.4 ± 17.1 s for the control and VR groups, respectively (Table 3). The resternotomy time target (resternotomy < 5 min) was reached in 100% (14) of the procedures in the control group and in 83% (10) of the procedures in the VR group, with a mean time of 211.0 ± 25.5 s and 273.0 ± 21.0 s for the control and VR groups, respectively. Both groups were significantly faster than the 5-min time target.

### 3.3. Secondary Outcomes

**Figure 3:**
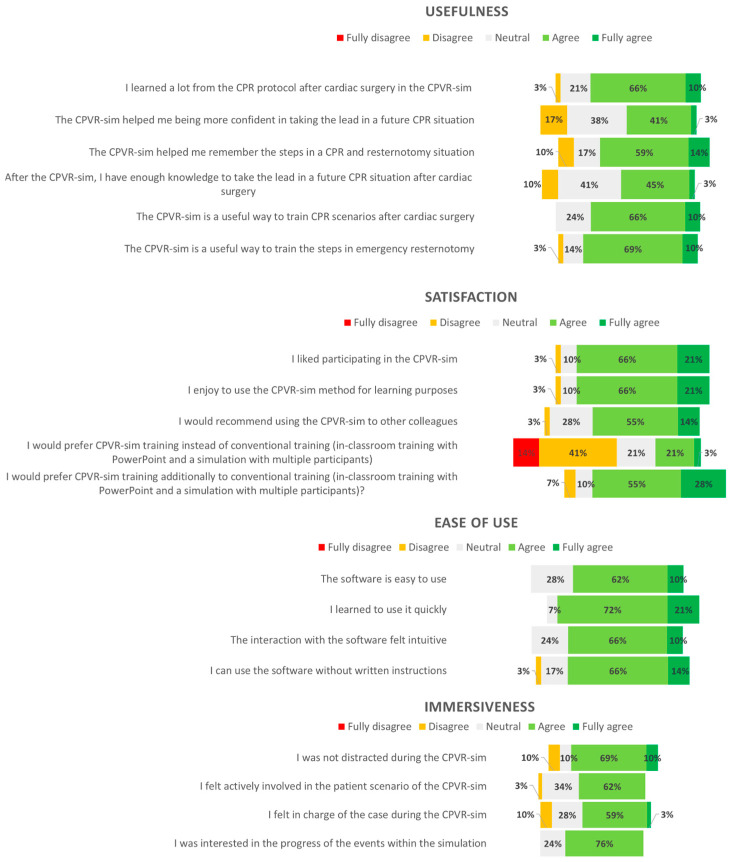
A diverging stacked bar chart demonstrating the usefulness, satisfaction, ease of use, and immersiveness data from participants rating the CPVR-sim. Inconsistencies in the sum of percentages is due to the rounding of the percentages. CPVR-sim—cardiopulmonary resuscitation virtual reality simulator.

In the VR group, the total amount of mistakes was lower than that in the control group (11 vs. 15 mistakes). However, the number of mistakes per patient case were comparable for both groups (control group 2 (0.8–3.3) versus VR group 2 (1–2)). Notably, most residents in both groups (57% (4) control group, 86% (6) VR group) failed to stop all running intravenous infusions (Table 3). Twenty-eight (100%) residents completed the USE questionnaire. Most participants, 76% (22) learned a lot from the CPVR-sim, and the simulation helped most (72%, n = 21) of the participants remember the steps required in a CPR situation. Most participants enjoyed the CPVR-sim (86%, n = 25), and enjoyed using VR simulations for learning purposes (86%, n = 25). According to the participants, the software was easy to use (72%, n = 21) and quick to learn (93%, n = 27), without needing written instructions (79%, n = 23); the results are depicted in Figure 3.

## 4. Discussion

In this randomized controlled trial, we included 28 CTS residents with more than one year of experience, which took place on a national training day organized by the Dutch association of thoracic surgery. We aimed to study the effectiveness of VR simulation training using the CPVR-sim, comparing it to conventional classroom CSU-ALS training in terms of the proportion of participants that achieved stacked shocks and resternotomy time targets. Previous randomized studies were performed on CPR training of laypersons using VR [9]. To the best of our knowledge, this was the first randomized trial of a VR-based CPR simulator designed specifically for ALS after cardiac surgery.

The primary findings were that both groups were significantly quicker in performing an emergency resternotomy versus the 5-min time target. However, in the control group, six (43%) participants were able to deliver the three stacked shocks within 1 min, compared to none in the VR group, who missed the target by an average of 25 s. Additionally, the control group was faster in performing most actions (Table 3). However, residents in the VR group made fewer errors during the assessment cases, suggesting that the CPVR-sim is a powerful and robust method for learning the correct steps during CPR after cardiac surgery.

We used the metric of time target to key steps, as performed in the study by Dunning et al. [4]. Additionally, time is a relevant metric given that the resuscitation guidelines emphasize that these timing targets to critical events such as shocks, BLS, and resternotomy ultimately improve outcomes [2]. None of the participants in the VR group were able to reach the shock time target, and only six participants (43%) of the control group reached this target. From this result, both training methods individually did not sufficiently prepare residents to administer shocks quickly enough to delay BLS for the allowed one min [2]. Additionally, the results suggest that although the VR-trained group was generally slower in the physical assessment, they completed the scenario with increased accuracy. Therefore, a combination of both training modalities, specifically an updated version of the CPVR-sim with an emphasis on timings followed by ALS, may help to ameliorate this delay, combining the strengths of both groups.

## 5. Limitations

Despite including all of the available CTS residents in the Netherlands, this remained a relatively small sample size. To logistically be able to fit all of the training and assessment cases into the single day that was assigned for us, it was necessary to divide the assessment cases into groups of two where one would perform the function of team leader and the other as the surgeon. As a result, we could not conduct a comparative analysis of the two groups.

The control group was more familiar with the moulage setting than the VR group, who did not engage with the manikin or any of the equipment until their assessment, likely incurring a delay whilst becoming oriented with their surroundings. This was reflected in the low number of shock time targets achieved by the VR group versus the comparable number of resternotomy time targets achieved relative to the control group. Additionally, the control group had the opportunity to practice scenarios more than once during their training before the assessment, whereas the VR group only completed each scenario once. Future study participants should have a chance to interact with the assessment equipment and environment before their assessment. This is a further limitation of this study.

## 6. Future Outlook

We propose further developing the CPVR-sim by emphasizing speed in the scenarios and adding time limits on performing specific actions. This may accelerate the residents’ performance, ensuring time targets are consistently reached.

Secondly, similar standardized studies on a larger scale are required to assess residents’ performance in a controlled and standardized manner with clearly defined assessment criteria [10]. We therefore propose further research with a larger study population and the same performance metrics. This could be performed with European or international CTS residents.

Other specific points of improvement of the CPVR-sim include reinforcing the concept that directly recharging the defibrillator after the first and second shocks saves crucial time. Then whilst recharging, a rhythm check can be performed concurrently, and the decision to give another shock can be made based on this rhythm check without wasting seconds waiting for the defibrillator to charge. This and several other improvements based on the participants’ feedback on this study have already been made, and future iterations of the simulator aim to ameliorate these issues. Given that both training methodologies appeared to not result in consistently quick performance, future studies should consider whether a combination of VR and conventional training methodologies results in a greater number of time targets being achieved, in accordance with the blended learning approach suggested by the ERC [5]. VR has the advantage of being consistent and standardized and does not vary per instructor or center, whereas this is not the case for ALS training, where the content may vary depending on personal preference.

There are unpublished cases in which the drug administration could possibly have led to the arrest, highlighting the importance of stopping any running infusions, as stated in the guidelines [2]. Since the residents did not stop the running infusions in 71% (n = 10) of the physical assessments, we would suggest emphasizing the importance of stopping the infusions during future training, both in the conventional classroom training and the VR simulation training.

Finally, the literature reveals that CPR performance deteriorates after training, especially for staff not routinely performing this procedure, which is also the case for CPR after cardiac surgery [11,12]. In neonatal resuscitation, it has been shown that extra simulation training after nine months is sufficient for knowledge and skill retention [13]. Therefore, we suggest that CPVR simulation may be additionally and regularly used to retrain and repeat the training independently of a trainer and equipment.

## 7. Conclusions

To the best of our knowledge, this was the first RCT simultaneously assessing VR simulation training with conventional training for CSU-ALS. Based on the results, we suggest focusing more on fast stacked shocks in future iterations of the VR simulator and further developing the simulator based on the participants’ feedback to make the product an even better adjunct tool to conventional training. To conclude, we have demonstrated that VR is an adequate additional method to sustain knowledge and skills for ALS in these infrequent situations.

## Figures and Tables

**Table 1:** Table showing demographic and baseline data for participants in each group. Data are presented as n (%).

Baseline Category	Virtual Reality n = 14		Conventional n = 14		*p*-Value
**Female, n (%)**	3	21%	4	29%	0.66 *
**Age, mean ± SD**	31.3	±1.9	32.3	±2.4	0.24 °
**Work experience, n (%)**					1.00 †
<4 years	8	57%	8	57%	
>4 years	6	43%	6	43%	
**Experience with CPR after cardiac surgery, n (%)**					0.54 †
Never	1	7%	0	0%	
1–5 times	6	43%	7	50%	
5–10 times	7	50%	6	43%	
>10 times	0	0%	1	7%	
**Experience with emergency resternotomies, n (%)**					1.00 *
Never	0	0%	0	0%	
1–5 times	11	79%	10	71%	
5–10 times	3	21%	4	29%	
>10 times	0	0%	0	0%	
**Experience with emergency resternotomies for cardiac arrest, n (%)**					1.00 *
Never	0	0%	0	0%	
1–5 times	12	86%	13	93%	
5–10 times	2	14%	1	7%	
>10 times	0	0%	0	0%	
**Gaming experience, n (%)**					0.72 †
Never	1	7%	2	14%	
Few times	11	79%	11	79%	
Regular use	2	14%	1	7%	
**VR experience, n (%)**					0.45 †
Never	6	43%	8	57%	
Few times	8	57%	6	43%	
Regular use	0	0%	0	0%	
**Experience with simulation training, n (%)**					0.47 †
Never	2	14%	1	7%	
Multiple times	11	79%	13	93%	
Certified simulation trainer	1	7%	0	0%	
**Experience with digital training, n (%)**					0.42 †
Never	2	14%	5	36%	
Few times	9	64%	7	50%	
Multiple times	3	21%	2	14%	
**Knowledge of international STS guidelines, n (%)**	13	93%	10	71%	0.33 *
**Experience with simulation** **training in VR, n (%)**	2	14%	2	14%	0.60 *

* Fisher’s exact test, ° *t*-test, † Chi squared test.

**Table 2:** Table showing the proportion of participants from each group that achieved the time targets for the Cardiac Surgery Unit Advanced Life Support (CSU-ALS) protocol, in addition to statistical comparison to the time target using a one-sample *t*-test. Data are presented as mean ± SD or median (IQR).

Time Targets	Control Group n = 14	VR Group n = 14	Time Target	Control Groupmin		VR Group	
				Mean Difference	*p*-Value *	Mean Difference	*p*-Value *
Third shock [s] <1 min	6 (43%)	0 (0%)	60	2.7	0.22	25.4	0.004
Sternal Retractor Open [s] <5 min	14 (100%)	10 (71%)	300	−89.0	<0.001	−27.0	0.025

* One sample T-test versus Time Target.

**Table 3:** Table shows each group’s time to defined actions in the Cardiac Surgery Unit Advanced Life Support (CSU-ALS) algorithm. Data are presented as mean ± SD or median (IQR).

CSU-ALS Actions	Control Group	VR Group
	n = 14	n = 14
**Number of deviations from protocol**	2.0 (0.8–3.3)	2.0 (1.0–2.0)
**Call resus team [s]**	12.0 (6.5–13.3)	18.0 (12.0–23.0)
**Call thoracic surgeon [s]**	39.0 ± 35.8	93.9 ± 24.5
**Recognition of Correct Protocol [s]**	9.0 (6.0–16.0)	15.0 (6.0–49.0)
**Stop all running infusions [s]**	71.0 (68.0–88.0)	69 (-)
**First shock [s]**	43.6 ± 7.5	65.1 ± 13.1
**Third shock [s]**	62.7 ± 8.9	85.4 ± 17.1
**BLS [s]**	71.1 ± 8.9	72.9 ± 39.5
**BVM Vent [s]**	89.1 ± 25.5	78.0 ± 35.8
**Amiodarone given [s]**	67.4 ± 31.6	93.1 ± 44.3
**Decision Resternotomy [s]**	78.4 ± 11.2	116.0 ± 31.5
**Incision [s]**	174.0 ± 23.2	241.0 ± 46.6
**Removal of Steel Wires [s]**	192.1 ± 25.2	267.7 ± 53.7
**Sternal Retractor in Place [s]**	201.0 ± 26.9	280.7 ± 55.6
**Sternal Retractor Open [s]**	211.0 ± 25.5	273.0 ± 21.0
**Internal Cardiac Massage/Cleared Space for paddles [s]**	214 (0.0–188.3)	308 (0.0–325.0)
**Internal Defib [s]**	239.0 (216.8–269.8)	299.0 (289.0–357.0)
**Final Rhythm Check [s]**	248.0 (227.5–281.0)	318.0 (301.0–372.0)

## Data Availability

The data presented in this study are available on request from the corresponding author. The data are not publicly available due to the relatively small numbers of participants and their right to preserve their anonymity.

## Supplementary Materials

The following supporting information can be downloaded at: https://www.mdpi.com/article/10.3390/jcdd10020067/s1, S1: The Consolidated Standards of Reporting Trials Flowchart; S2: The Consolidated Standards of Reporting Trials Checklist. S3: Baseline Characteristics Questionnaire; S4: Assessment case and form; S5: Outlier detection statistics and plots.
