# Supplementary material for: Virtual Reality Simulator versus Conventional Advanced Life Support Training for Cardiopulmonary Resuscitation Post-Cardiac Surgery: A Randomized Controlled Trial
Source: J Cardiovasc Dev Dis. 2023 Feb 4;10(2):67. doi: 10.3390/jcdd10020067 (PMC9962457; doi:10.3390/jcdd10020067)
Supplement: Supplementary file 1 [file jcdd-10-00067-s001.zip › jcdd-2177871-supplementary.pdf]

Supplementary Materials S1 – CONSORT Flow Diagram

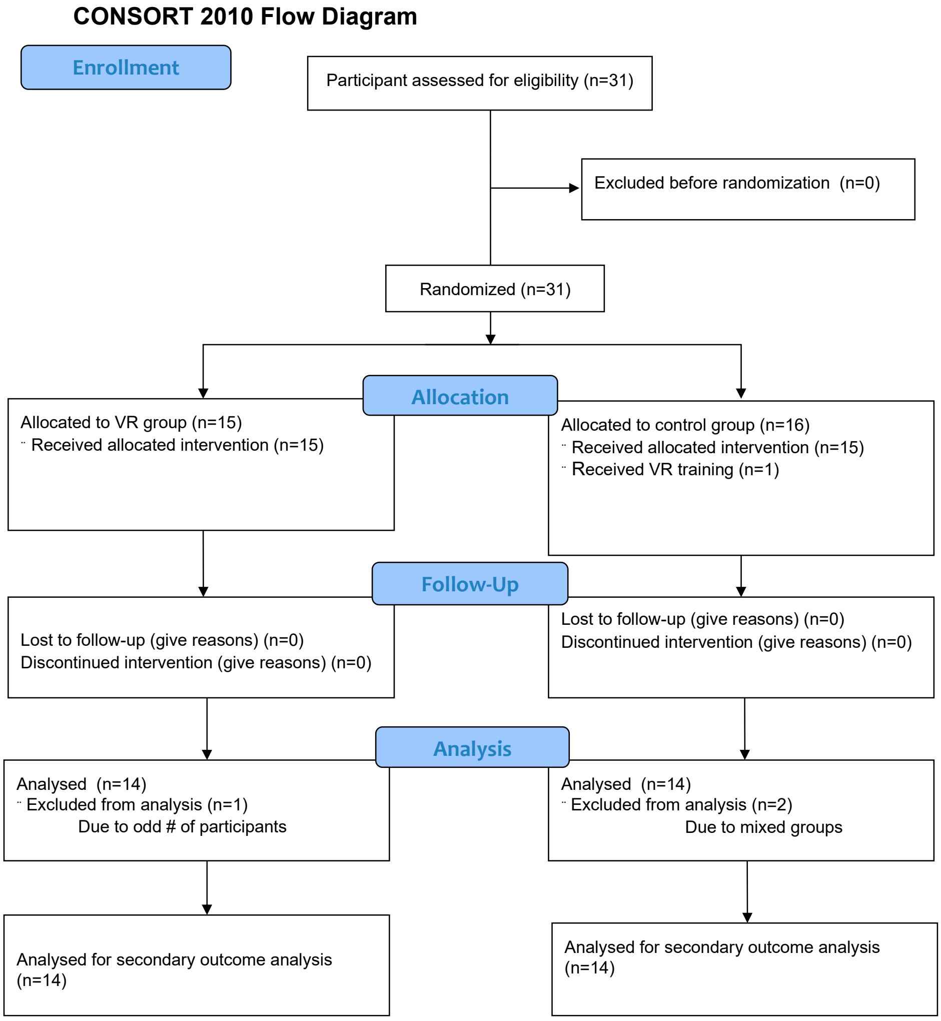

Supplementary Materials S2 – CONSORT Checklist

CONSORT 2010 checklist of information to include when reporting a randomised trial*

Section/Topic	Item No	Checklist item	Reported on page No
**Title and abstract**			
	1a	Identification as a randomised trial in the title	1
	1b	Structured summary of trial design, methods, results, and conclusions (for specific guidance see CONSORT for abstracts)	3
**Introduction**			
Background and objectives	2a	Scientific background and explanation of rationale	4
	2b	Specific objectives or hypotheses	5
**Methods**			
Trial design	3a	Description of trial design (such as parallel, factorial) including allocation ratio	5
	3b	Important changes to methods after trial commencement (such as eligibility criteria), with reasons	-
Participants	4a	Eligibility criteria for participants	5
	4b	Settings and locations where the data were collected	5
Interventions	5	The interventions for each group with sufficient details to allow replication, including how and when they were actually administered	6
Outcomes	6a	Completely defined pre-specified primary and secondary outcome measures, including how and when they were assessed	6
	6b	Any changes to trial outcomes after the trial commenced, with reasons	
Sample size	7a	How sample size was determined	5
	7b	When applicable, explanation of any interim analyses and stopping guidelines	
Randomisation:			
Sequence generation	8a	Method used to generate the random allocation sequence	5
	8b	Type of randomisation; details of any restriction (such as blocking and block size)	5
Allocation concealment mechanism	9	Mechanism used to implement the random allocation sequence (such as sequentially numbered containers), describing any steps taken to conceal the sequence until interventions were assigned	5,6
Implementation	10	Who generated the random allocation sequence, who enrolled participants, and who assigned participants to interventions	
Blinding	11a	If done, who was blinded after assignment to interventions (for example, participants, care providers, those	5

		assessing outcomes) and how	
	11b	If relevant, description of the similarity of interventions	6
Statistical methods	12a	Statistical methods used to compare groups for primary and secondary outcomes	7
	12b	Methods for additional analyses, such as subgroup analyses and adjusted analyses	7
**Results**			
Participant flow (a diagram is strongly recommended)	13a	For each group, the numbers of participants who were randomly assigned, received intended treatment, and were analysed for the primary outcome	7
	13b	For each group, losses and exclusions after randomisation, together with reasons	7
Recruitment	14a	Dates defining the periods of recruitment and follow-up	5
	14b	Why the trial ended or was stopped	
Baseline data	15	A table showing baseline demographic and clinical characteristics for each group	10
Numbers analysed	16	For each group, number of participants (denominator) included in each analysis and whether the analysis was by original assigned groups	7
Outcomes and estimation	17a	For each primary and secondary outcome, results for each group, and the estimated effect size and its precision (such as 95% confidence interval)	7,8
	17b	For binary outcomes, presentation of both absolute and relative effect sizes is recommended	7,8
Ancillary analyses	18	Results of any other analyses performed, including subgroup analyses and adjusted analyses, distinguishing pre-specified from exploratory	7,8
Harms	19	All important harms or unintended effects in each group (for specific guidance see CONSORT for harms)	
**Discussion**			
Limitations	20	Trial limitations, addressing sources of potential bias, imprecision, and, if relevant, multiplicity of analyses	8
Generalisability	21	Generalisability (external validity, applicability) of the trial findings	8,9
Interpretation	22	Interpretation consistent with results, balancing benefits and harms, and considering other relevant evidence	9
**Other information**			
Registration	23	Registration number and name of trial registry	1,5
Protocol	24	Where the full trial protocol can be accessed, if available	
Funding	25	Sources of funding and other support (such as supply of drugs), role of funders	1

*We strongly recommend reading this statement in conjunction with the CONSORT 2010 Explanation and Elaboration for important clarifications on all the items. If relevant, we also recommend reading CONSORT extensions for cluster randomised trials, non-inferiority and equivalence trials, non-pharmacological treatments, herbal interventions, and pragmatic trials. Additional extensions are forthcoming: for those and for up to date references relevant to this checklist, see www.consort-statement.org.

Supplementary Materials S3 - Baseline questionnaire

Participant # _____________

Baseline Characteristics Questionnaire

1. Gender: M / F / rather not say
2. Age: ____________
3. How many years of work experience do you have in cardiothoracic surgery working as a surgical resident? ____________

4. How many post-cardiac surgery resuscitation calls have you participated in?

□ I have never participated in a CPR situation
□ 1-5 times
□ 5-10 times
□ More than 10 times

5. How many emergency resternotomy procedures have you participated in?

□ I have never participated in an emergency resternotomy situation
□ 1-5 times
□ 5-10 times
□ More than 10 times

6. How many emergency resternotomy procedures for cardiac arrest have you participated in?

□ I have never participated in an emergency resternotomy situation
□ 1-5 times
□ 5-10 times
□ More than 10 times

7. Are you familiar with the international guidelines for cardiac arrest after cardiac surgery by the Society of Thoracic Surgeons?[1]

□ Yes
□ No

8. Do you have experience with gaming consoles (e.g. computer gaming, Xbox, PlayStation)?

□ I have never used a gaming console
□ I have used a gaming console a few times before
□ I am gaming on a regular basis (at least once a month)

9. How often do you use VR hardware/software (e.g. VR gaming, simulations, consoles, entertainment etc.)?

□ I have never had a VR experience until today
□ I have used VR a few times before
□ I am experienced and use VR on a regular basis (at least once a month)
□ I am an VR expert (have a VR console and applications myself)

10. Do you have experience with physical simulation trainings (e.g CPR or ALS trainings)?
- □ I have never had simulation training before
- □ I have had simulation trainings multiple times before
- □ I am a certified simulation trainer

11. Do you have experience with digital training (e.g. e-learning or serious games)?
- □ I have never had such training before
- □ I have had a digital training a few times before
- □ I have had digital trainings multiple times before

12. Do you have experience with simulation training in VR?
- □ Yes
- □ No

Supplementary Materials S4 - Assessment case and form

Case description (Dutch)

71-jarige vrouw, 3 dagen na een CABG met LIMA LAD, vene op de MO en RDP, voor non-STEMI bij drievats lijden. Patiënt heeft geen pacemakerdraden in situ. De telemetrie gaat af en laat breed complex tachycardie zien van 200/min. Jullie komen de patiënt kamer binnen, met een van de verpleegkundigen, en treffen patiënt bewusteloos aan. Wij geven aan wanneer de anesthesioloog in de kamer is, intubeert en sedeert. De leider heeft de leiding, de chirurg komt de kamer in zodra hij wordt gebeld door de leider, en zal steriel gaan staan met een van de verpleegkundigen.

Skill Assessment Form

Skill	Completed Correctly Yes	Completed Correctly No	Time to action (min:s)	Comments
**1. Recognition of the right protocol to use**				
2. Call resuscitation team				
3. Call thoracic surgeon				
4. Time to rhythm check				
5. Stopped all running infusions				
6. Time to first shock & third shock (in case of VF/VT)				
7. Time to initiate chest compressions (BLS)				
8. Time to initiate bag valve mask ventilation				
9. Time to first drug administration (if applicable)				
10. Time to first decision to open the chest				
11. Time to incision				
12. Removal of steel wires				
13. Sternal retractor in place				
14. Sternal retractor open				
15. Time to internal cardiac massage / clearing space for defibrillation paddles				
16. Time to internal defibrillation (in case of VF/VT)				
17. Final rhythm check				

Supplementary Materials S5 – Outlier Detection Statistics and Plots

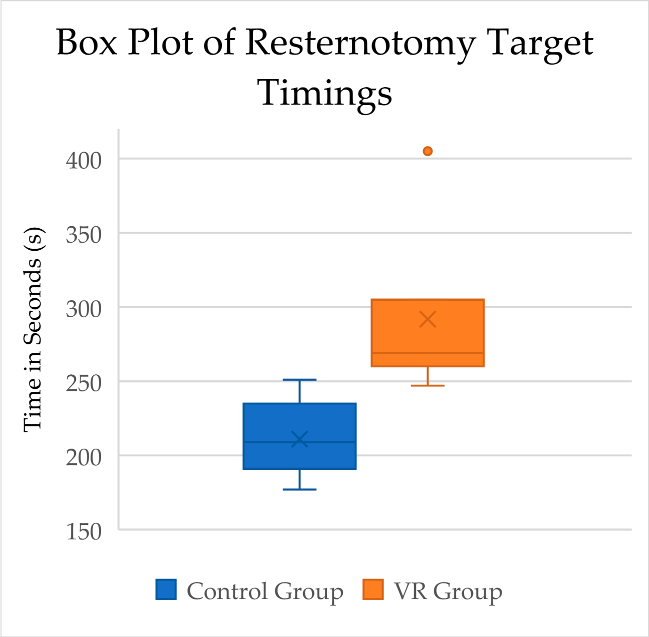

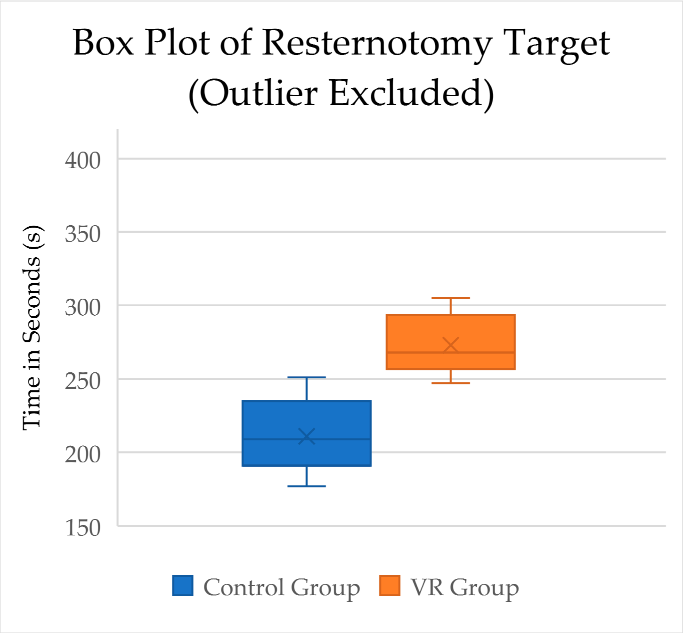

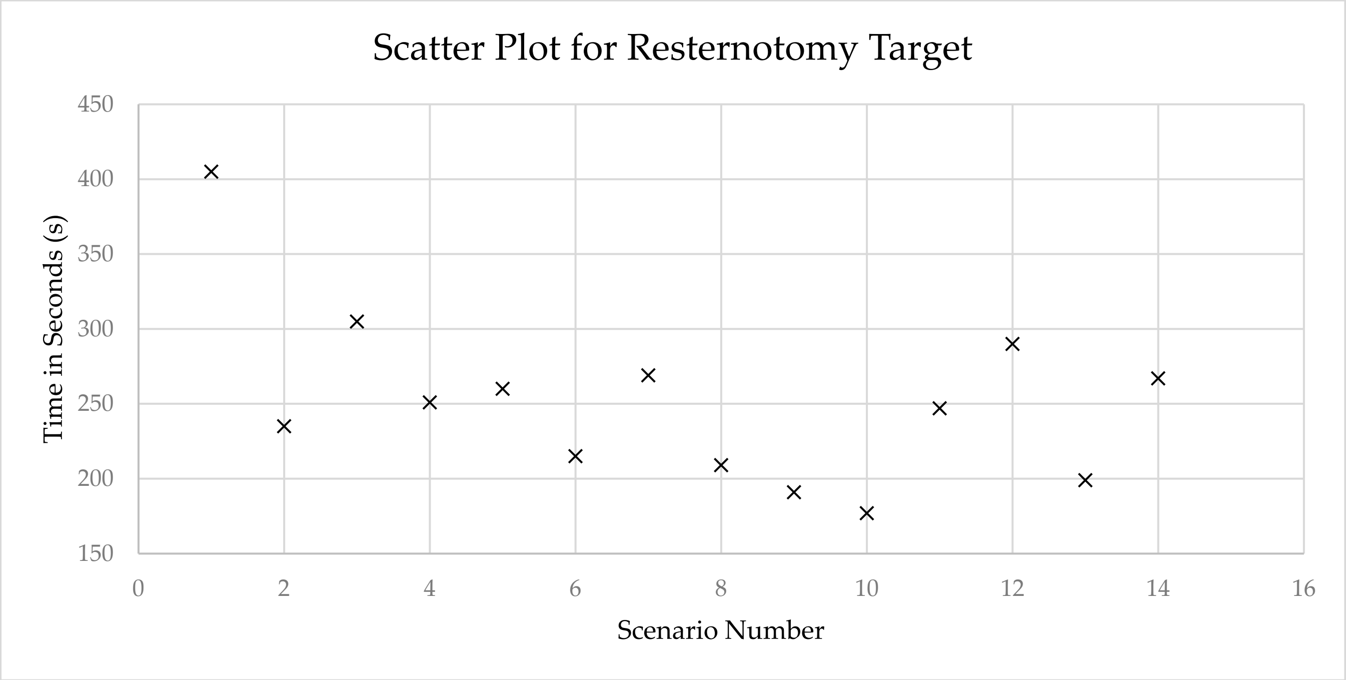

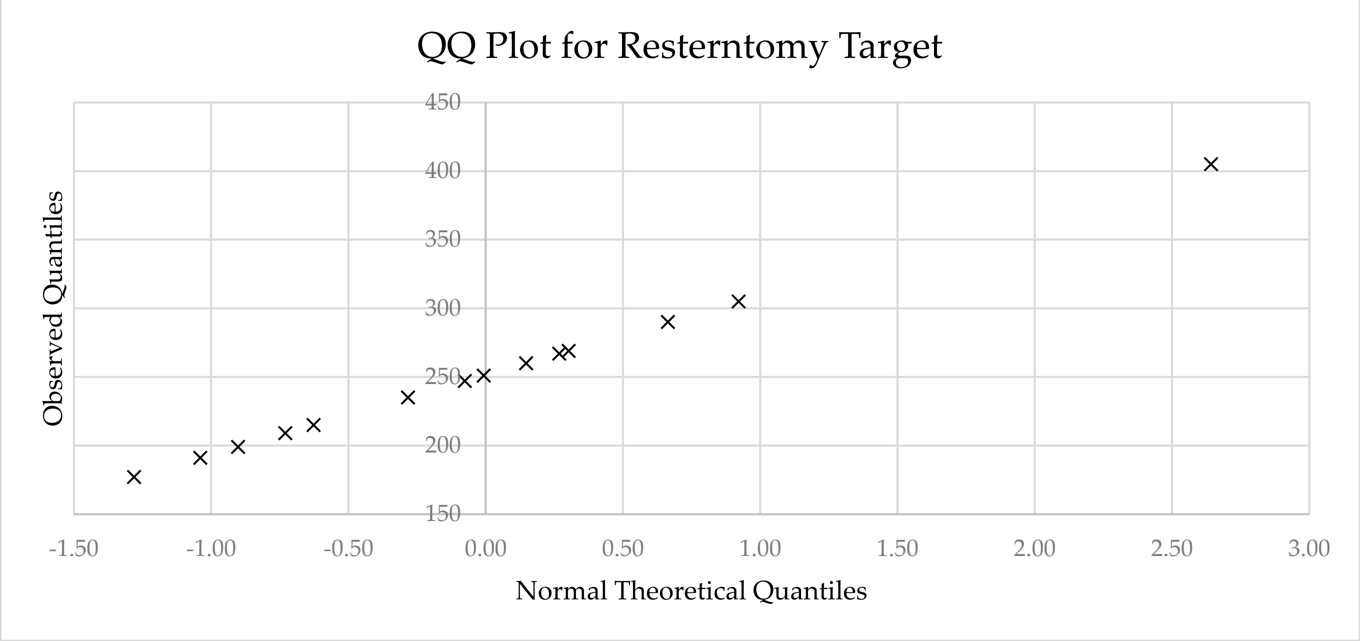

**Tukey's Fences Rule (1.5IQR Test)**	
Quartile 1 Value	210.5
Quartile 3 Value	268.5
Interquartile Range (IQR)	58
1.5 * IQR	87
Q3 + 1.5IQR	355.5
Data point in question	405
**Data point > Q3+1.5IQR**	**405>355.5**
**Outlier**	**Yes**

**Grubbs' Test**	
Maximum Value	405
Mean	251.428571
Standard Deviation	58.1268034
**Grubb's G**	**2.64200712**
Alpha	0.05
Sample Size	14
Significance value	0.00357143
Degrees of Freedom	12
t Critical Value	3.23573953
**G Critical Value**	**2.37165358**

**$G>G_{critical}$**	**2.64>2.37**
**Outlier**	**Yes**

**Dixon's Q**	
Q	0.885
Degrees of Freedom	13
Alpha	0.001
$Q_{ref\ (R21)}$	0.713
**$Q>Q_{ref}$**	**0.885>0.713**
**Outlier**	**Yes**
